# Prevalence of overweight and obesity and associated factors among persons with type 2 diabetes mellitus in Ethiopia: A systematic review and meta-analysis

**DOI:** 10.1016/j.obpill.2026.100313

**Published:** 2026-08-08

**Authors:** Samuel Semahgne Abrham, Mohammed Jemal, Baye Ashenef, Bitewlign Tiruneh, Matthias Kassie, Habtamu Belew, Ayenew Negesse

**Affiliations:** a Department of Human Nutrition, College of Medicine and Health Science, Debre Markos University, Debre Markos, Ethiopia; b Department of Biomedical Sciences, College of Medicine and Health Science, Debre Markos University, Debre Markos, Ethiopia; c Department of Medical Laboratory Sciences, College of Medicine and Health Science, Debre Markos University, Debre Markos, Ethiopia

**Keywords:** Diabetes mellitus, Overweight, Obesity, Prevalence, Meta-analysis, Ethiopia

## Abstract

**Introduction:**

The prevalence and associated factors of overweight and obesity among patients with type 2 diabetes mellitus (T2DM) were inconsistent in Ethiopia. Therefore, we aimed to pool the prevalence and associated factors of overweight and obesity among patients with T2DM using a systematic review and meta-analysis.

**Methods:**

This was a systematic review and meta-analysis of studies reporting overweight and obesity among patients with T2DM in Ethiopia. PubMed, MEDLINE, Scopus, Web of Science, Google and Google Scholar, and Cochrane Library databases were systematically searched for relevant studies from January 2015 to December 2025. In addition, a manual search was conducted using published articles’ reference lists. This review was conducted and reported in accordance with Preferred Reporting Items for Systematic Reviews and Meta-analyses (PRISMA) 2020 guideline. A narrative synthesis was provided by indicating the Adjusted Odds Ratio (AOR) with its corresponding 95% Confidence Interval (CI) of individual studies. All analyses were performed using STATA Version 17 software.

**Results:**

A total of 52 studies met the inclusion criteria. Of these, 47 studies contributed to the meta-analysis of prevalence, while six studies contributed to the narrative synthesis of associated factors. The pooled prevalence of overweight, obesity, and both overweight and obesity were 33.8% (95% CI: 30.7–37.0), 17.1% (95% CI: 13.6–20.6) and 51.1% (95% CI: 45.9–56.3), respectively. A significant heterogeneity was found across the included studies. Study region explained part of the observed heterogeneity. A narrative synthesis identified fifteen significant factors associated with overweight and obesity among patients with type 2 diabetes in Ethiopia. These factors were grouped into three categories: socio-demographic factors (age, sex, place of residence, educational level, monthly income and family history of overweight/obesity); behavioral factors (physical activity/exercise, dietary practice, alcohol consumption, walking status, walking level and vigorous physical activity); and clinical factors (comorbid hypertension, duration of diabetes, central obesity and triglyceride level).

**Conclusion:**

This study found that approximately half of patients with T2DM in Ethiopian studies were affected by overweight and obesity; there was a considerable difference across regions, with a higher prevalence observed in Addis Ababa. These findings highlight the importance of educating patients with T2DM on managing weight.

## Graphical abstract

**Figure:**
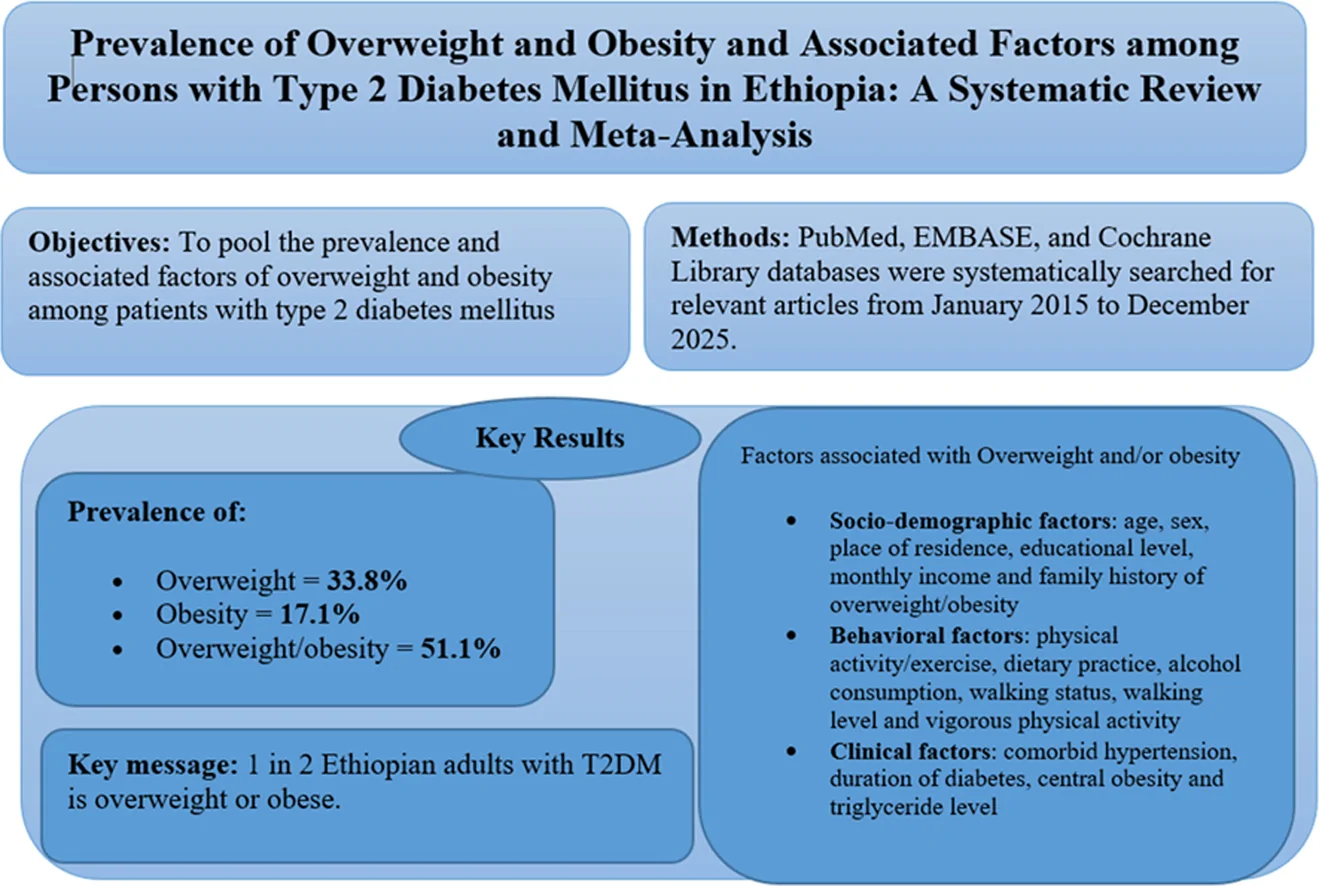

## 1 Introduction

Overweight and obesity have emerged as major global public health challenges contributing to chronic non-communicable diseases. They have risen to 2.5 billion in adults aged 18 years and older; obesity covers 890 million in the world [1]. Between 2000 and 2016, the number of overweight adults in Africa nearly doubled [2], and their rate nearly tripled over the last half century in Ethiopia [3].

The relation of obesity and diabetes, called as ‘‘diabesity’’ was researched extensively as a bidirectional relation. Obesity and overweight are well-established modifiable determinants of type 2 diabetes in the development, progression, and poor blood glucose level control [4,5]. Consequently, weight management is a standard of care for diabetes prevention and treatment [6]. The presence of type 2 diabetes leads to weight gain risk partly due to the anabolic effect of treatment and lifestyle changes [7]. Patients with type 2 diabetes with overweight and obesity improves glycaemia and reduce the need for glucose-lowering medications, particularly insulin, with modest weight loss [8,9].

In recent years, Ethiopia has experienced a rapid epidemiological transition characterized by changes in dietary patterns, sedentary lifestyles, urbanization, and lifestyle modifications. These changes lead to an increase in overweight, obesity, and chronic non-communicable diseases like type 2 diabetes [10,11]. While the country has been burdened by undernutrition, the coexistence of undernutrition and overnutrition, often referred to as ‘‘double burden of malnutrition’’ is now evident [12]. This dual challenge places additional strain, underscoring the need for context-specific evidence to inform policy and the health care system.

Studies have been conducted in different parts of Ethiopia about the prevalence of overweight and obesity among people with type 2 diabetes along with associated factors. Additionally, several studies are also available about complications, metabolic syndrome, dyslipidemia and comorbidities among people with type 2 diabetes who reported their weight status. Although these studies, they show considerable variability in their findings due to differences in the study setting, population characteristics, sample size and study design. As a result, the prevalence of overweight and obesity among persons with T2DM remains unclear, and the consistency of associated factors has not been systematically examined.

Therefore, this systematic review and meta-analysis aimed to estimate the pooled prevalence of overweight and obesity and to provide a narrative synthesis of associated factors among persons with type 2 diabetes mellitus in Ethiopia. The findings are expected to provide robust evidence to policymakers, program planners, and clinicians to strengthen integrated weight and diabetes management within the Ethiopian health care system.

## 2 Materials and methods

### 2.1 Registration and report of the study protocol

This was a systematic review and meta-analysis of studies reporting overweight and obesity among patients with type 2 diabetes mellitus in Ethiopia. This study was conducted in accordance with the Preferred Reporting Items for Systematic Reviews and Meta-Analyses (PRISMA) 2020 guideline [13]. The results were aligned with the standard PRISMA checklist (Additional File Table S1).

This systematic review and meta-analysis title, procedures and its protocol have been registered in the PROSPERO online database (with registration ID number CRD420261279684 and available from https://www.crd.york.ac.uk/PROSPERO/view/CRD420261279684.

### 2.2 Study setting

This systematic review and meta-analysis was conducted in Ethiopia.

### 2.3 Searching strategies

A systematic and comprehensive search of the literature was conducted to identify studies reporting the prevalence of overweight and obesity and their associated factors among patients with type 2 diabetes mellitus in Ethiopia. The databases searched were PubMed/MEDLINE, Scopus, Web of Science, and the Cochrane Library. In addition, grey literature was explored through Google and Google Scholar. Medical Subject Headings (MeSH) terms and relevant free-text keywords related to overweight and obesity, type 2 diabetes mellitus, and Ethiopia. The search terms were combined by Boolean operators (“AND” and “OR”) and the search strategy was adapted for each database as required. The search strategy was guided by the CoCoPop framework consisting of (Co)-condition, (Co)-context and (Pop)- population [14]: as (‘obesity’, ‘overweight’), (‘Ethiopia’), and (‘type 2 diabetes mellitus’) respectively. The article locating strategy was through (“overweight” OR “obesity” OR “body mass index” OR “BMI”) AND (“type 2 diabetes” OR “type II diabetes mellitus” OR “T2DM”) AND (“Ethiopia”). For studies that did not primarily focus on overweight and obesity we used “Metabolic Syndrome” OR “Metabolic Cardiovascular Syndrome” OR “Cardio-metabolic Syndrome” OR “Hypertension” OR ‘’High blood pressure’’ OR “Dyslipidemia” OR “Retinopathy’’ OR “Nephropathy” OR “Neuropathy” instead of (“overweight” OR “obesity” OR “body mass index” OR “BMI”).

This search strategy aimed to identify all relevant published and unpublished primary studies related to the topic. The selection was limited to studies published after the year 2015, in order to ensure that the most up-to-date and relevant studies were included. To identify additional eligible articles, the reference lists of included studies were also screened.

### 2.4 Study inclusion criteria

Cross sectional, cohort and case-control studies, studies involved adult patients with type 2 diabetes mellitus, studies conducted in Ethiopia, studies reported Body Mass Index (BMI) and categorized as overweight and obesity according to World Health Organization (WHO) standards—overweight (25–29.9 kg/m^2^) and obesity (≥30 kg/m^2^) [1]; and articles published in between 2015 and 2025.

Studies that assessed the prevalence and associated factors of overweight and obesity among patients with type 2 diabetes mellitus primarily were given priority for inclusion. However, studies that did not primarily focus on overweight and obesity but reported relevant data and met the other inclusion criteria were also included. For analysis of associated factors, only studies reported statistically significant adjusted associations with corresponding 95% confidence intervals (CIs).

The exclusion criteria were: reviews, editorials, commentaries, case reports, and conference abstracts without full data, animal research, and studies conducted in all types of diabetes where overweight and obesity were not reported separately for each type of diabetes.

### 2.5 The study selection and outcome

After the comprehensive literature search, all identified records were imported into the EndNote X9 reference management system software, and then duplicates were removed. Two independent reviewers (MJ, MK and TB) screened the remaining articles by using the settled inclusion criteria. Discrepancies between reviewers were resolved through discussion, and when necessary, other reviewers (SSA and HB) were consulted.

A second team of reviewers (BA and BT) evaluated the full-text articles for eligibility and methodological consistency before final inclusion. AN was the discrepancy resolver in this team during the study selection process. The predefined eligibility criteria, followed by documenting and reporting, were used for exclusion at the full-text screening stage. The overall process of study identification, screening, eligibility assessment, and inclusion was conducted in accordance with the PRISMA 2020 guidelines for systematic reviews and meta-analyses.

### 2.6 Risk of bias and methodological quality assessment

In accordance with PRISMA 2020 recommendations, the methodological quality of the included studies was critically appraised by using an appropriate tool (JBI critical appraisal tool) when assessing questions of incidence by three independent authors (AN, SSA, and MJ) [15]. The tool consists of 9 items designed to oversee the study population, sample size adequacy of the study, study subject and setting, reliability of condition or problem measurement, the appropriateness of the Statistical test used to analyze the data, and adequacy of the response rate of each selected article for the review process (Additional File Table S2).

Risk of bias was rated by questions of items that include an answer of “Yes”, “No”, or “Unclear” to rate its risk of bias (ROB). The inclusion and exclusion of screened articles was based on the overall quality appraisal score, in which an article was included if it scored equal to or above the average (4.5) of the two independent authors (BA and MK). Any disagreement during scoring the articles between the involved authors was solved through discussion, and the third author (SSA) was the arbiter.

Two independent authors (BT and SSA) extracted the data by using a standardized data extraction manual developed in 2014 by a Joanna Briggs Institute Reviewer [16]. Author name, year of publication, sample size, study region, study design, prevalence, and associated factors with overweight and obesity among type 2 diabetes, and adjusted odds ratios with 95% confidence intervals were information extracted from studies. Any inconsistency was cross-checked, and discrepancies raised during the data extraction process were resolved through discussion; if consensus could not be reached, a third author (AN) made the final decision.

### 2.7 Data synthesis and statistical analysis

All necessary and relevant information from every included study was extracted into a Microsoft Excel spreadsheet, and then exported to STATA Version 17 for further analysis. The primary outcomes of interest were the pooled prevalence of overweight, obesity, and overweight and obesity, as well as factors associated with overweight and/or obesity among patients with type 2 diabetes mellitus in Ethiopia. For each included study, the prevalence estimates and their corresponding standard errors were calculated.

The statistical heterogeneity among studies was assessed using Cochran's Q test and quantified using the I^2^ statistic, with I^2^ values of 25%, 50%, and 75% indicating low, moderate, and high heterogeneity, respectively [17]. Subgroup analyses were performed based on study period, sample size, and study region to explore potential sources of heterogeneity. Sensitivity analyses were conducted by sequentially removing individual studies to assess the robustness of the pooled estimates. Funnel plots and Egger's regression asymmetry test were used to evaluate publication bias, with a visual inspection for the former and a p-value <0.05 for the latter considered an indicator of potential publication bias.

Factors associated with overweight and obesity among patients with type 2 diabetes mellitus were narratively synthesized and summary table, as most variables could not be quantitatively pooled due to heterogeneity in categories.

## 3 Results

### 3.1 Search results

**Fig. 1:**
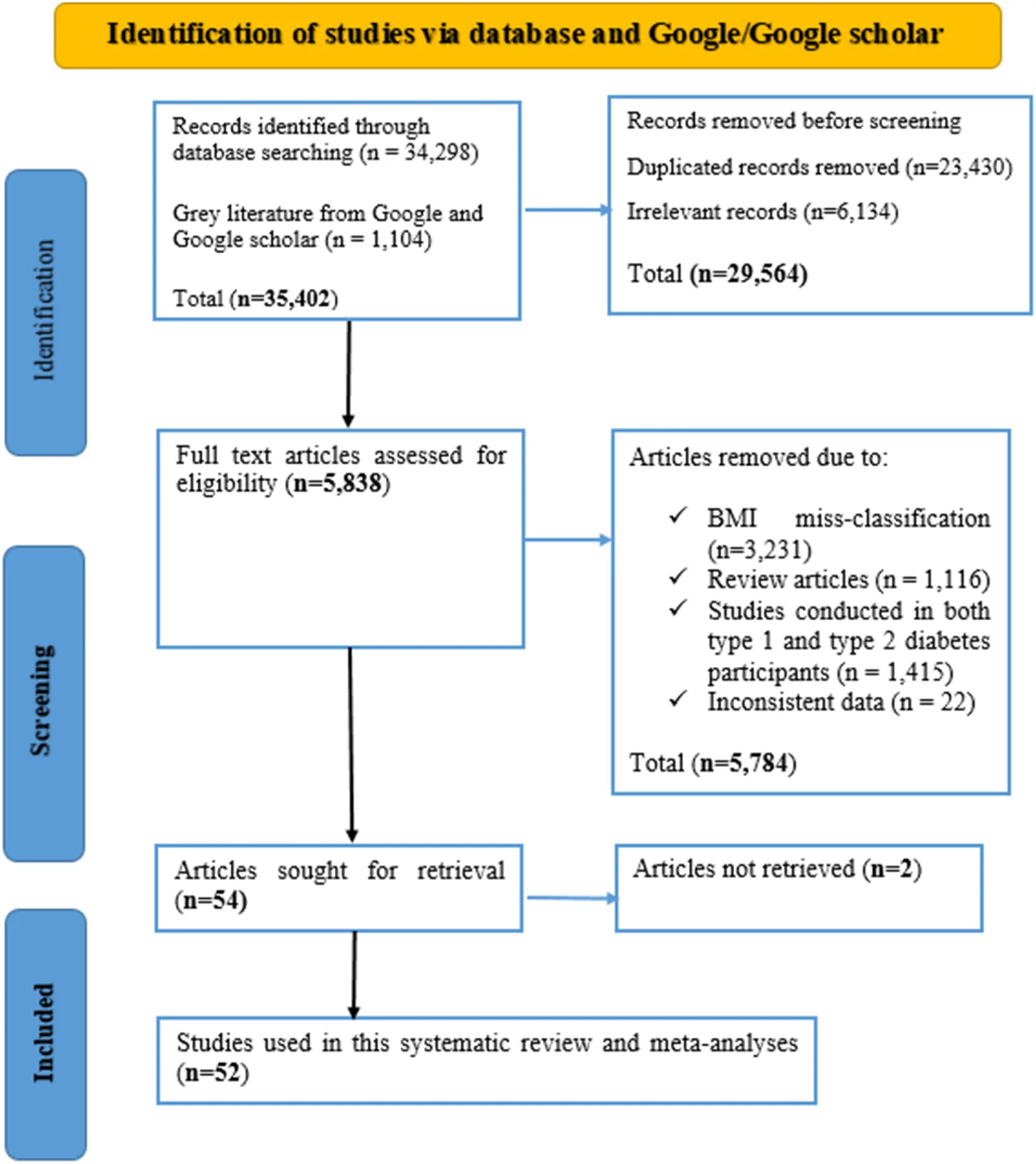
PRISMA flow diagram showing the study selection process for the systematic review and meta-analysis on overweight and/or obesity among patients with type 2 diabetes mellitus in Ethiopia.

A PRISMA flow diagram summarizing the study selection process is presented in Fig. 1. The initial literature search for this review was conducted from PubMed, MEDLINE, Scopus, Web of Science, and the Cochrane Library, yielding a total of 34,298 records. An additional 1104 records of grey literature was explored through Google and Google Scholar. Among the total of 35,402 records, 23,430 duplicates and 6134 irrelevant records were removed based on their abstract and title. Only 5838 remaining records were included for assessment; 54 articles were sought for retrieval and 52 articles were eligible to be included in the study. Of these, 47 articles were included in the meta analysis and 6 studies were included for factor analysis.

### 3.2 Characteristics of included studies

Only 5 studies aimed to assess the prevalence and determine associated factors of overweight/or obesity among patients with type 2 diabetes mellitus in Ethiopia [[18], [19], [20], [21], [22]]. One study assessed associated factors of overweight and obesity only [23]; the remaining 46 studies assessed other health problems/conditions but reported the BMI status of their participants [[24], [25], [26], [27], [28], [29], [30], [31], [32], [33], [34], [35], [36], [37], [38], [39], [40], [41], [42], [43], [44]]; [[45], [46], [47], [48], [49], [50], [51], [52], [53], [54], [55], [56], [57], [58], [59], [60], [61], [62], [63], [64], [65], [66], [67], [68], [69], [70]]. The highest number [16] of studies was conducted in Amhara, followed by Oromia [11], with the lowest study in Dire Dawa [1] and in the Afar region [1]. Only one study was conducted as a prospective observational study, four as case-control studies, and the remaining 47 were conducted in cross-sectional studies published between 2015 and 2025. This systematic review and meta-analysis study included a total of 17,517 patients with type 2 diabetes mellitus (Table 1).

**Table 1:** Characteristics of the included studies in the systematic review and meta-analysis on overweight and/or obesity among patients with type 2 diabetes mellitus in Ethiopia.

Authors name(Pub.year)	Region	Study Design	Overweight and/or obesity as the primary focus	Sample Size	Prevalence of overweight	Prevalence of obesity	Prevalence of overweight and/or obesity
Daba Abdissa (2021) [22]	Oromia	Cross sectional	Yes	334	29.3	6.9	36.2
Kbrom Gemechu Kiros (2019) [18]	Tigray	Cross sectional	Yes	365	40.8	NA	NA
Temesgen Bizuayehu (2023) [19]	Sidama	Cross sectional	Yes	314	36.3	18.8	55.1
Eshetu Elfios Endrias (2021) [20]	SNNPR	Cross sectional	Yes	526	31	18.9	49.9
Huzeyfa Kassaw(2024) [23]	Afar	Case control	Yes	286	NR	NR	NR
Abera Lambebo (2018) [21]	SNNPR	Cross sectional	Yes	407	30.7	4.4	35.1
Asamere Tsegaw (2021) [24]	Amhara	Cross sectional	No	739	34.98	10.6	45.6
Elias Mulat (2020) [25]	Oromia, Amhara, Sidama, Tigray, and Addis Ababa	Cross sectional	No	422	40.7	42.2	82.9
Aregash Abebayehu Zerga (2020) [26]	Amhara	Cross sectional	No	343	26.4	11.2	37.6
Worku Misganaw Kebede (2021) [27]	Amhara	Cross sectional	No	327	56	18	74
Ginenus Fekadu (2019) [28]	Oromia	Cross sectional	No	228	33.2	6.3	39.5
Tewodros Yosef (2021) [29]	Oromia	Cross sectional	No	245	35.1	19.8	54.9
Gudisa Bereda (2021) [30]	Oromia	Cross sectional	No	122	34.4	26.2	60.6
Rodas Getachew Abera (2022) [31]	Addis Ababa	Cross sectional	No	325	19.1	3.7	22.8
Mohammed Gebre Dedefo (2020) [32]	Oromia	Cross sectional	No	252	19.8	17.1	36.9
Tadele Eticha (2016) [33]	Tigray	Cross sectional	No	384	21.1	2.6	23.7
Berhane Fseha (2017) [34]	Tigray	Cross sectional	No	200	8	4	12
Zemenu Addis (2024) [35]	Amhara	Cross sectional	No	354	44.35	10.5	54.8
Tsion Shibru (2019) [36]	Addis Ababa	Cross sectional	No	191	43	24.7	67.7
Aster Wakjira Garedo (2024) [37]	Oromia	observational	No	420	26.67	54.8	81.4
Endalkachew Tedila Tarekegn (2025) [38]	Amhara	Cross sectional	No	465	21	17	38
Alemayehu Molla Tekalign (2025) [39]	Dire Dawa	Case control	No	190	31.57	24.7	56.3
Gebre Teklemariam Demoz (2019) [40]	Addis Ababa	Cross sectional	No	357	40.61	50.1	90.8
Ali Seid Kolbay (2024) [42]	Addis Ababa	Cross sectional	No	400	35.5	28	63.5
Gebrehiwot Lema Legese (2023) [43]	Amhara	Case control	No	180	39.4	16.1	55.5
Yitagesu Mamo (2019) [44]	Oromia	Case control	No	410	28.3	8.3	36.6
Yadelew Yimer Shibabaw (2023) [45]	Amhara	Cross sectional	No	407	31.45	7.13	38.6
Biniyam Demisse Andarge (2025) [46]	SNNPR	Cross sectional	No	404	46	18.6	64.6
Mohammed Hassen Salih (2024) [47]	Amhara	Cross sectional	No	627	28.7	12.4	41.2
Gebreamlak Gebremedhn Gebremeskel (2019) [48]	Tigray	Cross sectional	No	419	26.96	8.4	35.3
Agete Tadewos (2017) [56]	Sidama	Cross sectional	No	270	34.1	11.1	45.2
Abel Shita (2023) [58]	SNNPR	Cross sectional	No	204	35.3	17.6	52.9
Belete Biadgo (2018) [49]	Amhara	Cross sectional	No	159	44	11.3	55.3
Girma Deshimo Lema (2024) [50]	Amhara	Cross sectional	No	380	38.9	7.1	46
Rukiya Debalke (2020) [51]	Oromia	Cross sectional	No	422	35.7	19.6	55.3
Mulatua Yeshitla (2025) [52]	Addis Ababa	Cross sectional	No	310	48.4	18.4	66.8
Wabi Temesgen Atinafu (2025) [53]	Addis Ababa	Cross sectional	No	420	16.3	14.8	31.1
Misganaw Asmamaw Mengstie (2024) [54]	Oromia	Cross sectional	No	124	26.6	7.3	33.9
Mitku Mammo Taderegew (2021) [55]	Amhara	Cross sectional	No	366	26.2	12.5	38.7
Firegenet Asnake Kitaw (2023) [57]	Addis Ababa	Cross sectional	No	314	43.0	26.4	69.4
Kidist Tamru (2020) [59]	Addis Ababa	Cross sectional	No	346	41.62	37.3	78.9
Kehabtimer S Kotiso (2020) [60]	SNNPR	Cross sectional	No	365	45.2	13.9	59.1
Fikreab Desta (2024) [61]	Oromia	Cross sectional	No	378	46.6	5.3	51.9
Leteslase Hagos Gebreziher (2024) [62]	Addis Ababa	Cross sectional	No	417	35.3	8.5	43.8
Haftay Gebremedhin (2020) [63]	Tigray	Cross sectional	No	420	13.6	0.2	13.8
Zemenu Addis (2025) [64]	Amhara	Cross sectional	No	416	41.6	6.5	48.1
Samuel Agegnew Wondm (2024) [65]	Amhara	Cross sectional	No	413	15.3	31.7	47
Alebachew Ferede Zegeye (2023) [66]	Amhara	Cross sectional	No	496	56.3	17.3	73.6
Fasil Bayafers Tamene (2025) [67]	Amhara	Cross sectional	No	409	27.1	21.1	48.2
Tilaye Arega Moges (2025) [68]	Amhara	Cross sectional	No	398	46.5	38.9	85.4
Berhanu Kelemework (2024) [69]	Sidama	Cross sectional	No	228	31.6	31.6	63.2
Tsion Habtamu Ababiya (2023) [70]	Addis Ababa	Cross sectional	No	270	44.4	9.3	53.7

### 3.3 Prevalence of overweight

**Fig. 2:**
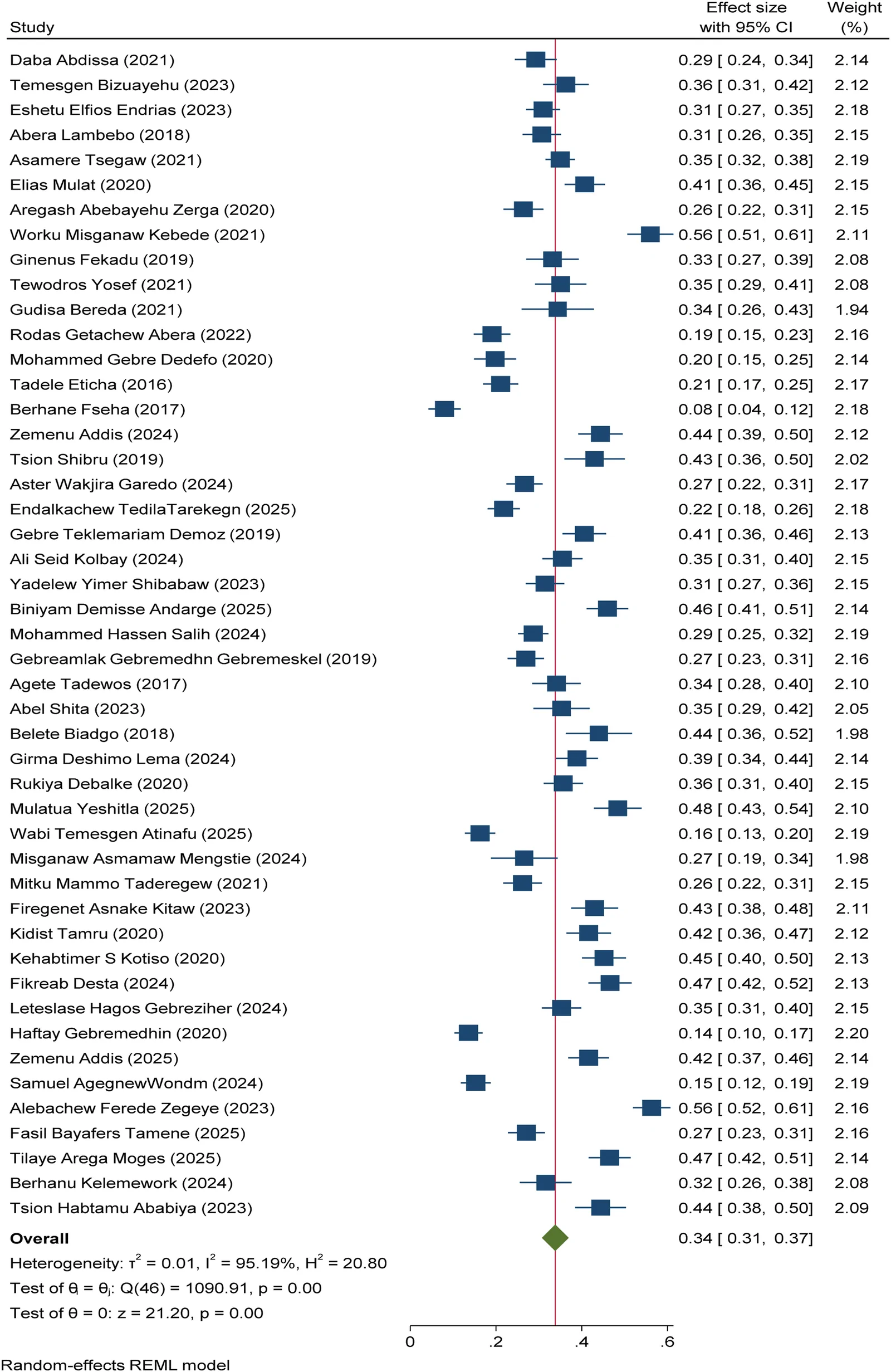
Forest plot of the pooled prevalence of overweight among patients with type 2 diabetes in Ethiopia.

Forty-seven studies reported the prevalence of overweight among patients with type 2 diabetes. The pooled prevalence of overweight was 33.8% (95 CI: 30.7–37.0). The reported prevalence ranges from 8.0% [34] to 56.3% [66]. However, substantial heterogeneity was observed among the included studies (I^2^ = 95.19%, p < 0.001), as shown in Fig. 2. The 95% prediction interval ranged from 12.2% to 55.5%, indicating that the true prevalence in a future similar population may plausibly fall within this range.

### 3.4 Prevalence of obesity

**Fig. 3:**
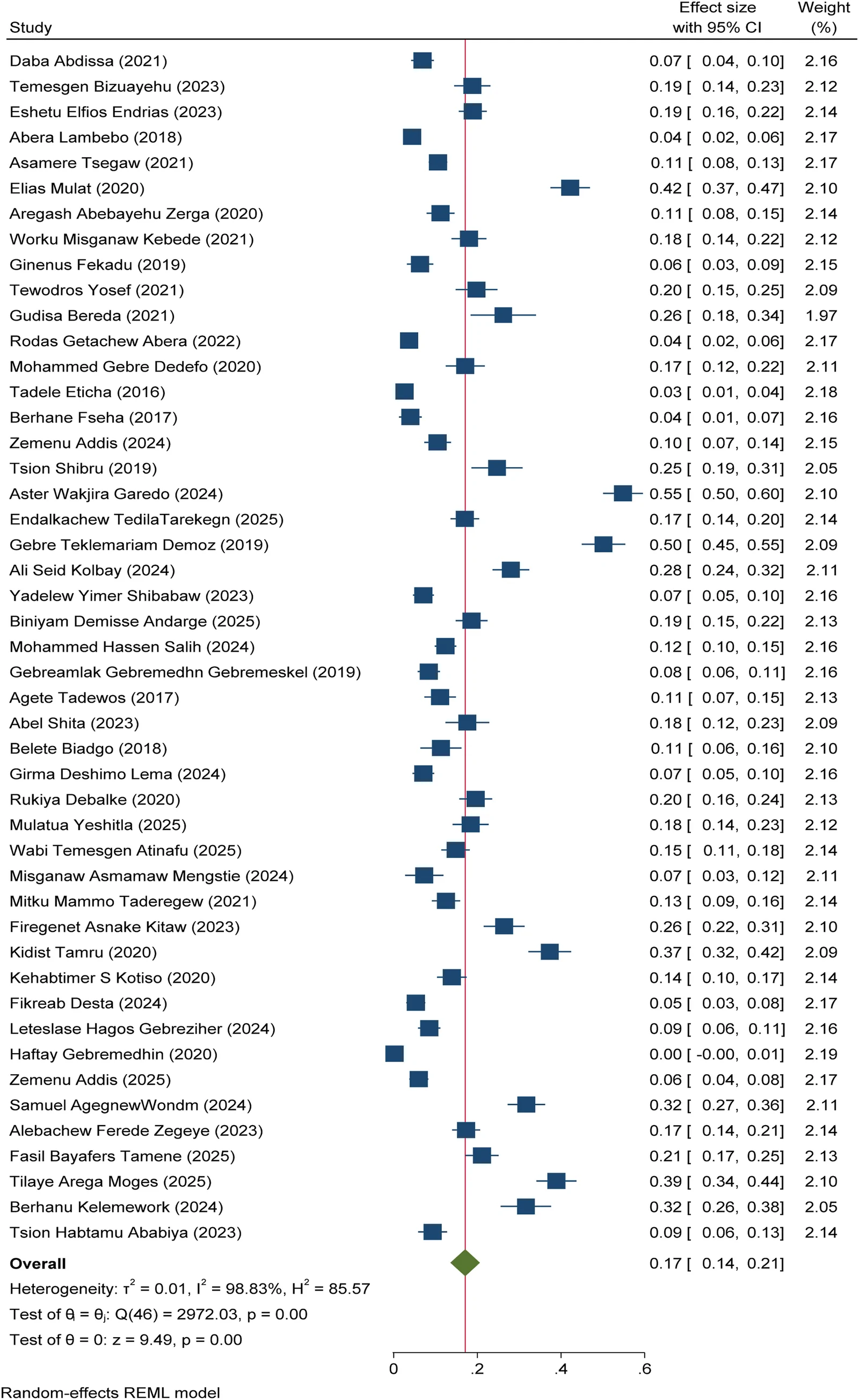
Forest plot of the pooled prevalence of obesity among patients with type 2 diabetes in Ethiopia.

Similar to overweight, forty-seven studies reported the prevalence of obesity as one, two, or three grades; if so summed up as total obesity. The pooled prevalence of obesity was 17.1% (95 CI: 13.6–20.6). The prevalence ranged from 0.2% [63] to 54.8% [37]. The 95% prediction interval ranged from 7.7% to 41.9% since high heterogeneity was observed among the studies (I^2^ = 98.83%, p < 0.01), as shown in Fig. 3.

**Fig. 4:**
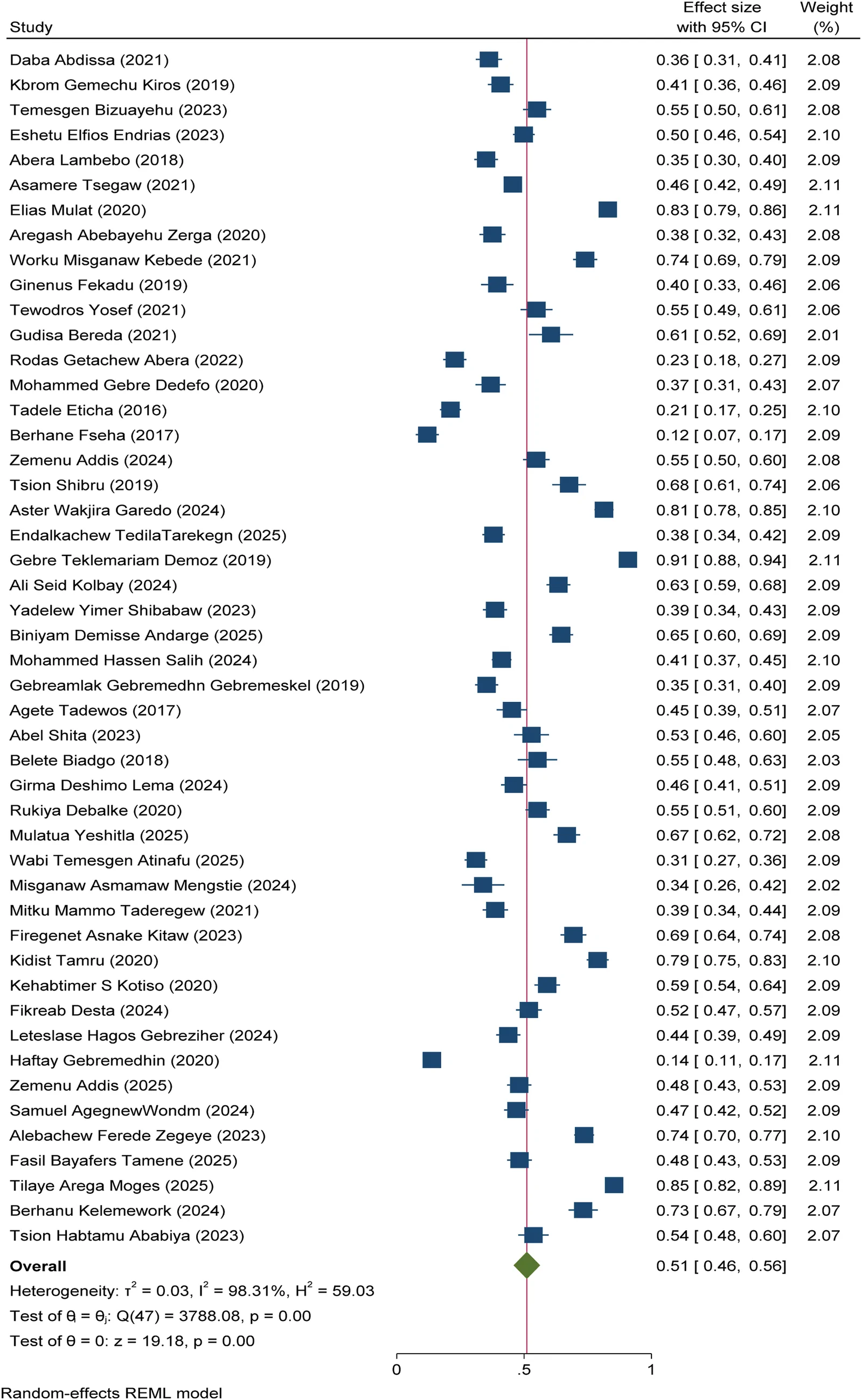
Forest plot of the pooled prevalence of overweight and obesity among patients with type 2 diabetes in Ethiopia.

### 3.5 Prevalence of overweight and obesity

The prevalence of both overweight and obesity was determined from forty-eight studies. The pooled prevalence of overweight and obesity was 51.1% (95 CI: 45.9–56.3). The prevalence ranged from 12.0% [34] to 90.8% [40]. However, substantial heterogeneity was observed among the included studies (I^2^ = 98.31%, p < 0.01), as shown in Fig. 4. The 95% prediction interval ranged from 13.9% to 88.3%, indicating that the true prevalence in a future similar population may fall within this range.

### 3.6 Subgroup **and** meta-regression analysis

The pooled prevalence of overweight and obesity were used for the sub group and meta-regression analysis. We conducted subgroup analysis on the basis of the study region, sample size and study period by national nomenclatures of 13 regions and 2 administrative cities, < and ≥ mean sample size(350) and studies conducted before 2020 and 2020 and above.

The pooled prevalence of overweight and obesity did significantly differ by the study region, sample size and study period. The study shows that the highest pooled prevalence of overweight and obesity was observed studies published during and after 2020 (53.8%), sample size less than 350 (51.3%) and studies conducted in Addis Ababa 58.9%. One study was conducted in multiple regions(Oromia, Amhara, Sidama, Tigray and Addis Ababa) reported that the prevalence of overweight and obesity were 82.9% [25] and not included in the regional subgroup analysis (Table 2).

**Table 2:** Subgroup analysis of studies the systematic review and meta-analysis on overweight and/or obesity among patients with type 2 diabetes mellitus in Ethiopia.

Sub group criteria	Sub group	Number of studies	Prevalence
Study period	<2020	20	47.3% (95 CI: (37.9–56.7))
	≥2020	28	53.8% (95 CI: (47.9–59.7))
Mean sample size	<350	20	51.3% (95 CI: (43.4–59.3))
	≥350	28	50.9% (95 CI: (43.9–58.0))
Study region	Tigray	5	24.5% (95 CI: (13.3–35.8))
	Amhara	15	51.5% (95 CI: (43.9–59.1))
	Oromia	9	50.2% (95 CI: (40.0–60.3))
	SNNPR	5	52.3% (95 CI: (42.4–62.2))
	Sidama	3	57.8% (95 CI: (41.8–73.9))
	Addis Ababa	10	58.9% (95 CI: (45.7–72.0))

We conducted analysis of meta-regression using study year, sample size, study region for prevalence as covariates to explore potential sources of heterogeneity, so study region explained part of the observed of heterogeneity (Table 3).

**Table 3:** Meta-regression analysis of heterogeneity among studies on overweight and/or obesity among patients with type 2 diabetes mellitus in Ethiopia.

	Source of heterogeneity	Coefficient	Standard error	P value
Sub group of study	Study period	0.0645664	0.0537823	0.230
	Sample size	−00.0038856	0.0546758	0.943
	Study region	0.0172418	0.0055997	0.002

### 3.7 Publication bias

**Fig. 5:**
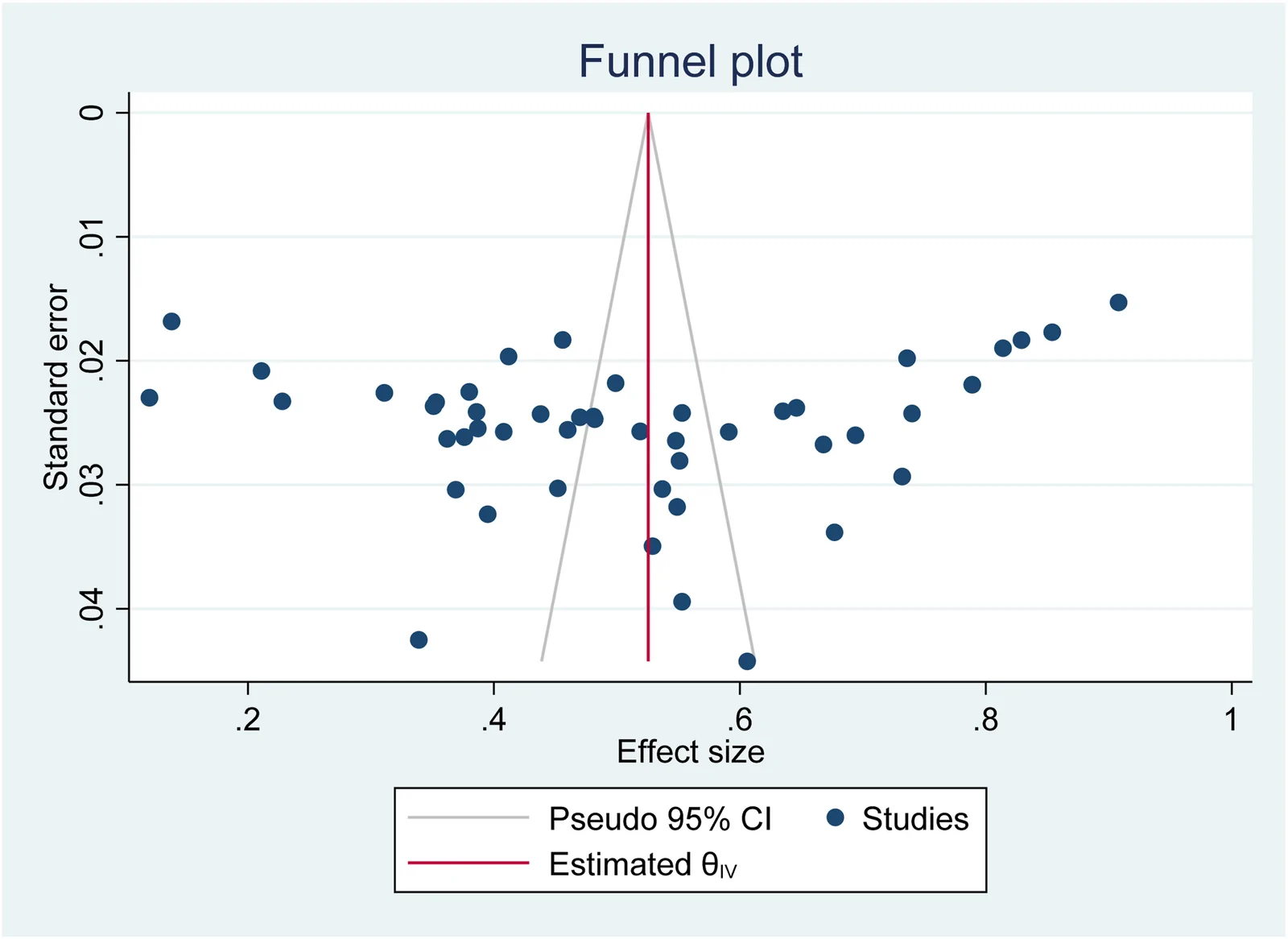
Funnel plot test for prevalence of overweight and obesity among patients with type 2 diabetes in Ethiopia.

Publication bias was assessed using the funnel plot and the regression-based Egger test for the test of small study effect. Although the funnel plot suggested asymmetry in visual inspection, the regression-based Egger test conducted under a random-effects model did not indicate any evidence of small study effects (β_1_ = −2.46, SE = 4.51, z = −0.55, p = 0.585) (Fig. 5).

### 3.8 Sensitivity analysis

**Fig. 6:**
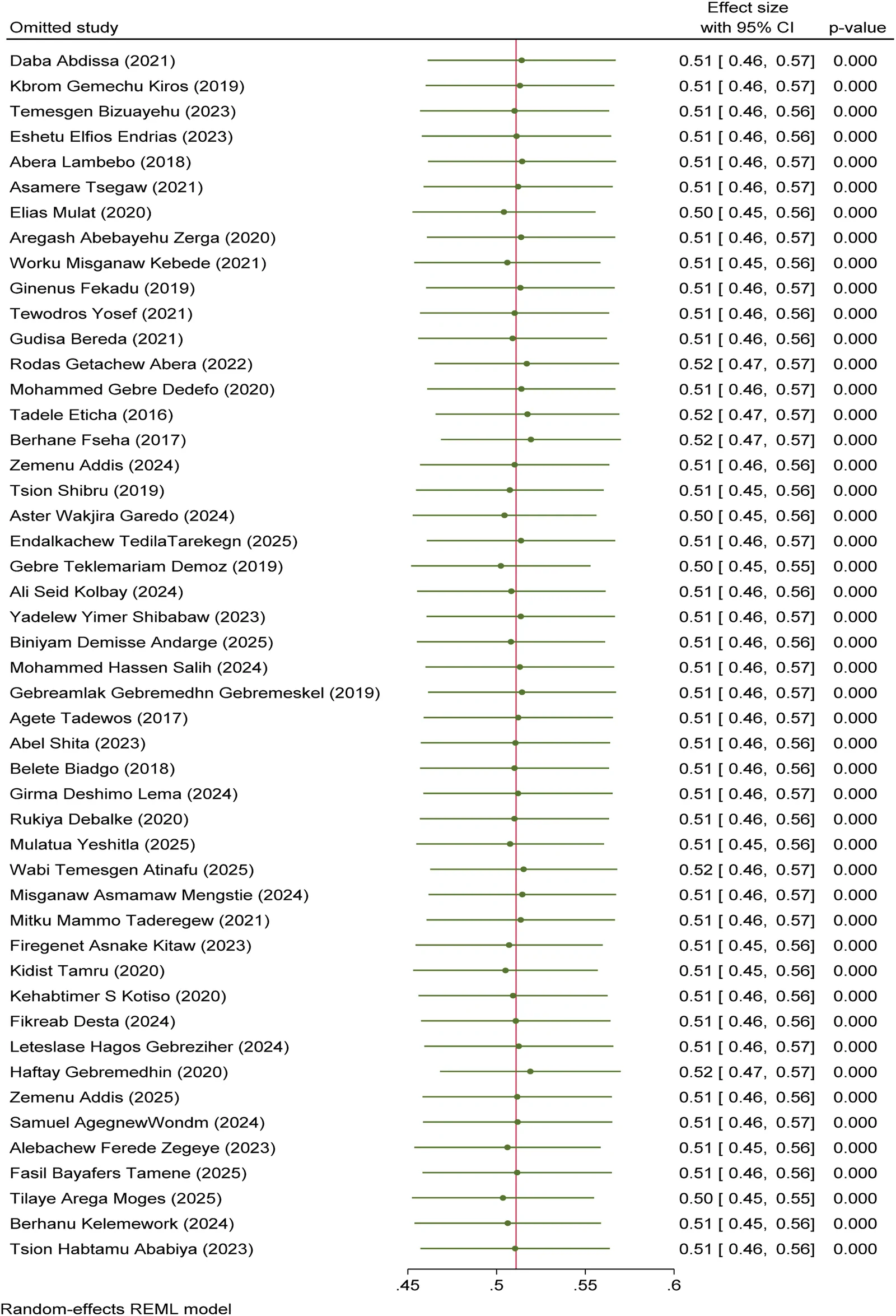
Sensitivity analysis on prevalence of overweight and obesity among patients with type 2 diabetes in Ethiopia.

We also conducted a sensitivity analysis by removing one study at a time to examine the influence of each study on the pooled result, but the omission of any single study did not materially alter the pooled effect estimate on the overall effect size (Fig. 6).

### 3.9 Factors associated with overweight and obesity

Six studies assessed factors associated with overweight and obesity among patients with type 2 diabetes mellitus in Ethiopia and met the eligibility criteria for this review, and all provided adjusted effect measures in the form of odds ratio (OR) [[18], [19], [20], [21], [22], [23]].

Overall, 15 factors were reported to be significantly associated with overweight and obesity among patients with T2DM. These factors were grouped into three categories: socio-demographic factors (age, sex, place of residence, educational level, monthly income and family history of overweight/obesity); behavioral factors (physical activity/exercise, dietary practice, alcohol consumption, walking status, walking level and vigorous physical activity); and clinical factors (comorbid hypertension, duration of diabetes, central obesity and triglyceride level). Table 4 presents a summary of all factors significantly associated with overweight and obesity.

**Table 4:** Summary of the factors associated with overweight and obesity among patients with T2DM in Ethiopia.

Factors	N studies	Author	Significant response	Adjusted results, AOR (95% CI)
**Socio-demographic factors**				
Age in years	1	Abera Lambebo (2018)	45–54 ≥55	2.41 (1.34–4.31) 3.24(1.74–6.06)
Sex	2	Temesgen Bizuayehu (2023)	Female	3.00 (1.60–5.70)
		Eshetu Elfios Endrias(2023)		1.98 (1.35–2.90)
Residence	2	Daba Abdissa (2021)	Urban	1.80 (1.00–3.00)
		Kbrom Gemechu Kiros (2019)		3.40 (1.26–9.40)
Educational level	1	Huzeyfa Kassaw (2024)	College and above	10.30 (4.16–25.50)
Monthly income	1	Daba Abdissa (2021)	≥58.9 USD	3.40 (1.80–6.70)
Family history of overweight/obesity	1	Daba Abdissa (2021)	Yes	1.90 (1.10–3.40)
**Behavioral factors**				
Physical activity/exercise	3	Daba Abdissa (2021)	Inactive	2.10 (1.20–3.50)
		Eshetu Elfios Endrias(2023)	Low	3.10 (1.72–5.61)
		Abera Lambebo (2018)	<30 min daily	2.31 (1.30–4.11)
Dietary practice	3	Kbrom Gemechu Kiros (2019)	Poor	8 (4.02–15.5)
		Abera Lambebo (2018)	Poor	2.98 (1.79–4.96)
		Eshetu Elfios Endrias(2023)	Poor	1.964 (1.347–2.864)
Alcohol consumption	2	Kbrom Gemechu Kiros (2019)	Yes	2.9 (1.5–5.5)
		Eshetu Elfios Endrias(2023)	2–3/week	2.442 (1.315–4.432)
Walk	1	Kbrom Gemechu Kiros (2019)	No	2.3 (1.01–5.17)
Walk level			Low	3.3 (1.45–7.61)
Vigorous activity	1	Kbrom Gemechu Kiros (2019)	Low	4 (1.19–13.8)
**Clinical factors**				
Comorbid HTN	2	Daba Abdissa (2021)	Yes	2.40 (1.40–4.00)
		Eshetu Elfios Endrias(2023)	Yes	1.54 (1.04–2.30)
Duration of Diabetes	2	Kbrom Gemechu Kiros (2019)	3–6 years	2.8 (1–7.85)
		Abera Lambebo (2018)	3–6 years	2.55(1.48–4.40)
Central obesity	1	Kbrom Gemechu Kiros (2019)	Yes	3.4 (1.64, 6.91)
TG level	1	Temesgen Bizuayehu (2023)	≥200 mg/dl	3.6(1.6–8.3)

### 3.10 Socio-demographic factors

Age, sex, place of residence, educational level, monthly income and family history of overweight/obesity were identified as significant factors associated with overweight and obesity in patients with T2DM. One study reported that patients with T2DM with age of 45–54 and ≥ 55 years were more than two and more than three times likely to be overweight and obese as compared to those with age 25–44 years (AOR 2.41, 95% CI: 1.34–4.31) and (AOR 3.24, 95% CI: 1.74–6.06)) respectively [21].

Two studies identified a significant association between overweight and obesity and being female. Both studies found that being female increased the odds of being overweight and obesity. One study reported that female patients were almost twice as likely to be overweight and obese as compared to male patients (AOR 1.98, 95% CI: 1.35–2.90) [20]. The other study reported an even stronger association, with female patients having three times the odds of being overweight and obese as compared to male patients (AOR = 3.00, 95% CI: 1.60–5.70) [19].

Again, two studies found a significant association between overweight and obesity and residences. Both studies found patients with T2DM with urban residence were more likely associated with overweight and obesity, with the strength of association ranging from (AOR = 1.80, 95% CI: 1.00–3.00) [22] to (AOR = 3.40, 95% CI: 1.26–9.40) [18].

Only one study reported educational status were significantly associated with overweight and obesity. Educational status of college and above were more than ten times the odds of being overweight and obesity (AOR = 10.30, 95% CI: 4.16–25.50) [23]. Similarly, one study found that monthly income were significantly associated with overweight and obesity. The study reported patients with T2DM with average monthly income(AMI) of greater or equal of 58.9 United States Dollars (USD) having more than three times the odds of being overweight and obese as compared to those with AMI below 29.5 USD (AOR 3.4, 95% CI: 1.8–6.7) [22].

Family history of overweight or obesity was reported as a significant factor for being overweight and obese in one study. The study found that patients with a family history of overweight or obesity had almost more than 2 times the odds of being overweight and obese as compared with their counterparts (AOR 1.90, 95% CI: 1.10–3.40) [22].

### 3.11 Behavioral factors

Low physical activity or being physically inactive was reported as associated factor for being overweight and obese among patients with type 2 diabetes in Ethiopia in three studies [[20], [21], [22]]. One study reported that patients with T2DM with physically inactive status were more than two times were more than twice as likely to be overweight and obese as compared to those with physically active patients (AOR 2.10, 95% CI: 1.20–3.50) [22]. Another study found that the odds of being overweight and obese were more than three times higher in patients with low physical activity than their counterparts (AOR 3.10, 95% CI: 1.72–5.61) [20]. The third study also reported that patients who engaged in physical activity for less than 30 min per day had more than twice the odds of being overweight or obese compared with those who engaged in physical activity for at least 30 min per day (AOR 2.31, 95% CI: 1.30–4.11) [21].

Three studies found that poor dietary practice was significantly associated with being overweight or obese. Patients with poor dietary practice had approximately twofold, threefold, and eightfold higher odds of being overweight or obese than those with good dietary practice (AOR = 1.96, 95% CI: 1.35–2.86(20); AOR = 2.98, 95% CI: 1.79–4.96(21); and AOR = 8.00, 95% CI: 4.02–15.50(18), respectively).

Two studies found that alcohol consumption were significantly associated with being overweight or obese. The first study reported that patients who consumed alcohol had almost three times higher odds of being overweight or obese than who did not consume alcohol (AOR 2.90, 95% CI: 1.50–5.50) [18]. Another study indicated that the odds of being overweight or obese were more than twice in patients who consumed alcohol 2-3x/week than who never consumed (AOR 2.44, 95% CI: 1.32–4.43) [20].

Walk status and level reported as a significant factor for overweight or obesity in one study. The study found that patients who did not walk were more than two times higher odds of being overweight or obese than who walked (AOR 2.30, 95% CI: 1.01–5.17). This study also found that patients with low level of walk had more than three higher odds of being overweight or obesity than patient who walk in adequate level (AOR 3.30, CI: 1.45–7.61). This study again reported that patient with low vigorous activity had four times higher odds of being overweight or obesity than patient who engaged in adequate vigorous activity [18].

### 3.12 Clinical factors

Hypertension was reported as a significant factor associated with overweight and obesity among patients with T2DM in two studies. One study found that patients with comorbid hypertension had more than twice the odds of being overweight or obese compared with those without hypertension (AOR = 2.40, 95% CI: 1.40–4.00) [22]. Similarly, another study reported that patients with comorbid hypertension had 1.54 times higher odds of being overweight or obese than those without hypertension (AOR = 1.54, 95% CI: 1.04–2.30) [20].

Two studies identified the duration of diabetes as a significant factor associated with overweight and obesity. Both studies reported that patients with a diabetes duration of 3–6 years had higher odds of being overweight or obese than those with a shorter duration. The reported adjusted odds ratios were 2.80 (95% CI: 1.00–7.85) [18] and 2.55 (95% CI: 1.48–4.40) [21].

One study reported that central obesity was significantly associated with overweight and obesity among patients with T2DM. Patients with central obesity had more than three times higher odds of being overweight or obese than those without central obesity (AOR = 3.40, 95% CI: 1.64–6.91) [18]. Another study found that elevated triglyceride levels were significantly associated with overweight and obesity. Patients with triglyceride levels of ≥200 mg/dL had 3.60 times higher odds of being overweight or obese than those with triglyceride levels below 200 mg/dL (AOR = 3.60, 95% CI: 1.60–8.30) [19].

## 4 Discussion

This systematic review and meta-analysis synthesized evidence from studies conducted on diverse topics and settings about the prevalence and narratively synthesized associated factors of overweight and obesity among patients with T2DM in Ethiopia. The prevalence of overweight and obesity was 33.8% and 17.1%, respectively. This study showed a higher prevalence of obesity(5.44%) as compared to the general adult population in the country [71]. This indicates that T2DM might be an indicator of obesity. As compared to studies conducted among patients with T2DM in Germany and Jordan, 50.9% and 58.6%, respectively, this study found a lower prevalence [72,73]. The discrepancy may be due to differences in socioeconomic status, levels of physical activity, cultural practices, urbanization, dietary patterns, and genetic factors.

The pooled prevalence of overweight and obesity was 51.1%. The wide prediction intervals indicate considerable between-study variability and suggest that the prevalence may vary substantially across future Ethiopian populations. The prevalence was slightly low as compared with the study conducted in Africa level 61.4% and subgroup analysis in East Africa, 56.9% [74]. Higher prevalence was also reported as 77.9% 85.3% in in France and the United Kingdom, respectively. Beyond this lowest prevalence, there is an increasing rate of overweight and obesity due to nutrition transition and sedentary lifestyle [75].

Studies conducted in recent years ( ≥ 2020) in sub-group analysis found slightly higher prevalence than their counterparts. This may reflect ongoing urbanization, economic growth, and lifestyle changes**,** which have intensified exposure to obesogenic environments over time. The change in sample size for the study did not significant difference in prevalence according to study.

Furthermore, we observed that the prevalence of overweight and obesity was varied across different regions, with the highest prevalence in Addis Ababa, 58.9%. This finding may be attributed to urban living conditions, including increased availability of processed foods, reduced physical activity, higher sedentary behavior, and lifestyle changes associated with modernization. Urban residents may also experience greater exposure to unhealthy dietary patterns and lower levels of occupational and recreational physical activity compared with rural populations.

In terms of associated factors, this study narratively synthesized 15 factors that were reported to be significantly associated with overweight and obesity among patients with T2DM.

The identified factors were categorized into socio-demographic, behavioral, and clinical factors. Socio-demographic factors comprised older age, being female, urban residence, college and above educational level, monthly income of greater or equal of 58.9 USD and having family history of overweight/obesity. Behavioral factors included low or no physical activity/exercise, poor dietary practice, alcohol consumption, no walking, low walking levels and low level vigorous activity. Clinical factors included comorbid hypertension, longer duration of diabetes, central obesity and ≥200 mg/dL triglyceride level.

Most of these findings are biologically and behaviorally plausible and align with existing evidence [1,76,77]. Reduced physical activity and metabolic changes with aging, combined with gender-specific social and cultural roles, may partly explain the higher risk observed among older adults and women. Additionally, the coexistence of hypertension and obesity highlights the clustering of cardio-metabolic risk factors among patients with type 2 diabetes.

### 4.1 Limitation of the study

Although a comprehensive search strategy and large pooled sample are on the side of the strength of this systematic review acknowledge several limitations. First, the majority of included studies were cross-sectional in design, which limits inferring causal relationships between overweight and obesity and the identified associated factors. Second, substantial heterogeneity was observed across studies, even after conducting subgroup analyses and meta-regression. Although study region explained a part of the observed heterogeneity, considerable residual heterogeneity remained, which may be attributable to unmeasured differences in study populations, measurement methods, diagnostic criteria, and healthcare settings. Consequently, this analysis is limited by extreme statistical heterogeneity (I^2^ > 95%), indicating substantial variation among studies. Therefore, the pooled estimates should be interpreted cautiously and considered descriptive rather than definitive.

Third, in all included studies body mass index (BMI) used to assess overweight and obesity. BMI does not capture central adiposity, nor does distinguish between fat mass and lean mass, which may lead to misclassification of adiposity, particularly among patients with T2DM. Fourth, several included studies did not primarily focus on overweight and obesity but reported BMI as a secondary outcome, which may have resulted in variability in data quality and measurement rigor. Fifth, limited number of studies was used for associated factor synthesis, potentially excluded. Six the possibility of publication bias cannot be completely ruled out, although no statistical evidence of publication bias was detected using Egger's test and visual asymmetry in the funnel plot. Seven, this study will be limited in representation from rural areas and some regions of Ethiopia, which may affect the generalizability to all patients with type 2 diabetes mellitus nationwide. Finally, the synthesis of associated factors included only statistically significant adjusted associations reported by the primary studies. Studies reporting non-significant adjusted associations were not synthesized, which may have introduced selective reporting bias and could have overestimated the consistency of the reported associations.

## 5 Conclusion

Overall, the findings of this systematic review and meta-analysis emphasize that overweight and obesity are highly prevalent among patients with type 2 diabetes in Ethiopia. A narrative synthesis identified several socio-demographic, behavioral and clinical factors. These results highlight the need for integrated interventions**,** including lifestyle modification, promotion of physical activity, and context-specific nutritional counseling, particularly in urban settings.

Clinical Takeaway Messages:

• Screening and routine assessment of overweight and obesity among individuals with type 2 diabetes mellitus should be integrated into diabetes care services, since overweight and obesity are common problems among individuals with type 2 diabetes mellitus in Ethiopia.
• Lifestyle interventions, including physical activity promotion and nutrition counseling, should be strengthened as essential components of type 2 diabetes management.
• Targeted prevention and management strategies should consider socio-demographic, behavioral, and clinical factors associated with overweight and obesity to address high-risk groups.

## Ethical approval

Ethical approval was not required for this study because it was a systematic review and meta-analysis of previously published studies and did not involve primary data collection from human participants or animals. Informed consent was not applicable.

## Data availability

The data used to conduct this study are available from the corresponding author upon request.

## Clinical trial number

Not applicable.

## Author contribution

SSA: Methodology, Software, Supervision, Formal Analysis, Writing – original draft, Writing – review & editing. HB: Visualization, Writing – review & editing, Formal Analysis, Investigation. MJ: Writing – original draft, Writing – review & editing, Visualization, Data Curation. BA: Visualization, Writing – review & editing, Formal Analysis, Investigation. BT: Writing – review & editing, Conceptualization, Methodology. MK: Writing – original draft, Visualization, Investigation. AY: Writing – review & editing, Conceptualization, Methodology, Supervision.

## Declaration AI use

The authors used ChatGPT 5 to assist in the linguistic domain for clarity, conciseness and readability during preparation of the original manuscript. All AI-assisted content was reviewed, edited, and verified by the human authors, who take full responsibility for the integrity of the work.

## Funding

No funding was received.

## Conflict of interest

The authors reported no potential conflicts of interest.

## Abbreviations

AOR –: Adjusted Odds Ratio
BMI –: Body Mass Index
CI –: Confidence Interval
PRISMA –: Preferred Reporting Items for Systematic Reviews and Meta-Analyses
T2DM –: Type 2 Diabetes Mellitus
WHO –: World Health Organization
