# Supplementary material for: Prevalence of overweight and obesity and associated factors among persons with type 2 diabetes mellitus in Ethiopia: A systematic review and meta-analysis
Source: Obes Pillars. 2026 Aug 8;19:100313. doi: 10.1016/j.obpill.2026.100313 (PMC13507751; doi:10.1016/j.obpill.2026.100313)
Supplement: Supplementary file 1 [file mmc1.docx]

**Table S 1: Methodological Quality assessment of the included primary studies**

| Author | Q1 | Q2 | Q3 | Q4 | Q5 | Q6 | Q7 | Q8 | Q9 | Score /9 |
| --- | --- | --- | --- | --- | --- | --- | --- | --- | --- | --- |
| Birarra MK, Gelayee DA.[16] | Y | Y | Y | Y | Y | Y | Y | Y | Y | 9 |
| Bizuayehu Wube T et al. [17] | Y | Y | Y | Y | Y | Y | Y | Y | Y | 9 |
| Tadewos A. et al.[21] | Y | Y | N | Y | Y | Y | Y | Y | Y | 8 |
| Zerga AA, Bezabih AM. [18] | Y | Y | N | Y | Y | Y | Y | Y | Y | 9 |
| Belete B. et al.[26] | Y | Y | N | Y | Y | Y | Y | Y | Y | 8 |
| Gemeda, D., E., et al.[22] | Y | Y | Y | N | Y | Y | Y | Y | Y | 8 |
| Charkos and Getnet.[25] | Y | UN | Y | Y | N | Y | N | Y | Y | 7 |
| Shita et al.[19] | Y | Y | Y | Y | Y | Y | Y | Y | Y | 9 |
| Woyesa et al.[23] | N | Y | Y | Y | Y | Y | N | Y | Y | 8 |
| Getachew Abebe.[24] | Y | Y | N | N | Y | Y | Y | Y | UN | 7 |
| Gebremeskel et al.[20] | Y | Y | Y | Y | Y | Y | Y | Y | Y | 9 |

N=No, NA= Not Applicable, UN= Unclear, Y=Yes,

Q1: Was the sample frame appropriate to address the target population? Q2: Were study participants sampled appropriately? Q3: Was the sample size adequate? Q4: Were the study subjects and the setting described in detail? Q5: Was the data analysis conducted with sufficient coverage of the identified sample? Q6: Were valid methods used for the identification of the condition? Q7: Was the condition measured in a standard, reliable way for all participants? Q8: Was there an appropriate statistical analysis? Q9: Was the response rate adequate and if not, was the low response rate managed appropriately?
