# Supplementary material for: Prevalence of overweight and obesity and associated factors among persons with type 2 diabetes mellitus in Ethiopia: A systematic review and meta-analysis
Source: Obes Pillars. 2026 Aug 8;19:100313. doi: 10.1016/j.obpill.2026.100313 (PMC13507751; doi:10.1016/j.obpill.2026.100313)
Supplement: Supplementary file 2 [file mmc2.docx]

| **Section and Topic** | **Item #** | Checklist item | **Location where item is reported** |
| --- | --- | --- | --- |
| TITLE | | |  |
| Title | 1 | **Prevalence of Overweight and Obesity and Associated Factors among Persons with Type 2 Diabetes Mellitus in Ethiopia: A Systematic Review and Meta-Analysis** |  |
| ABSTRACT | | |  |
| Abstract | 2 | Abstract  **Introduction:** The prevalence and associated factors of overweight and obesity among patients with type 2 diabetes mellitus (T2DM) were inconsistent in Ethiopia. Therefore, we aimed to pool the prevalence and associated factors of overweight and obesity among patients with T2DM using a systematic review and meta-analysis.  **Methods:** This was a systematic review and meta-analysis of studies reporting overweight and obesity among patients with T2DM in Ethiopia. PubMed, MEDLINE, Scopus, Web of Science, Google and Google Scholar, and Cochrane Library databases were systematically searched for relevant studies from January 2015 to December 2025. In addition, a manual search was conducted using published articles’ reference lists. This review was conducted and reported in accordance with Preferred Reporting Items for Systematic Reviews and Meta-analyses (PRISMA) 2020 guideline. A narrative synthesis was provided by indicating the Adjusted Odds Ratio (AOR) with its corresponding 95% Confidence Interval (CI) of individual studies. All analyses were performed using STATA Version 17 software.  **Results:** A total of 52 studies met the inclusion criteria. Of these, 47 studies contributed to the meta-analysis of prevalence, while six studies contributed to the narrative synthesis of associated factors. The pooled prevalence of overweight, obesity, and both overweight and obesity were 33.8% (95% CI: 30.7-37.0), 17.1% (95% CI: 13.6-20.6) and 51.1% (95% CI: 45.9-56.3), respectively. A significant heterogeneity was found across the included studies. Study region explained part of the observed heterogeneity. A narrative synthesis identified fifteen significant factors associated with overweight and obesity among patients with type 2 diabetes in Ethiopia. These factors were grouped into three categories: socio-demographic factors (age, sex, place of residence, educational level, monthly income and family history of overweight/obesity); behavioral factors (physical activity/exercise, dietary practice, alcohol consumption, walking status, walking level and vigorous physical activity**); and clinical factors (comorbid hypertension, duration of diabetes, central obesity and triglyceride level).**  **Conclusion:** This study found that approximately half of patients with T2DM in Ethiopian studies were affected by overweight and obesity; there was a considerable difference across regions, with a higher prevalence observed in Addis Ababa. These findings highlight the importance of educating patients with T2DM on managing weight.  **Keywords:** diabetes mellitus, overweight, obesity, prevalence, meta-analysis, Ethiopia |  |
| INTRODUCTION | | |  |
| Rationale | 3 | Overweight and obesity have emerged as major global public health challenges contributing to chronic non-communicable diseases. They have risen to 2.5 billion in adults aged 18 years and older; obesity covers 890 million in the world(1). Between 2000 and 2016, the number of overweight adults in Africa nearly doubled (2), and their rate nearly tripled over the last half century in Ethiopia(3). |  |
| Objectives | 4 | The relation of obesity and diabetes, called as ‘‘diabesity’’ was researched extensively as a bidirectional relation. Obesity and overweight are well-established modifiable determinants of type 2 diabetes in the development, progression, and poor blood glucose level control (4, 5). Consequently, weight management is a standard of care for diabetes prevention and treatment(6). The presence of type 2 diabetes leads to weight gain risk partly due to the anabolic effect of treatment and lifestyle changes(7). Patients with type 2 diabetes with overweight and obesity improves glycaemia and reduce the need for glucose-lowering medications, particularly insulin, with modest weight loss(8, 9). |  |
| METHODS | | |  |
| Eligibility criteria | 5 | Study inclusion criteria  Cross sectional, cohort and case-control studies, studies involved adult patients with type 2 diabetes mellitus, studies conducted in Ethiopia, studies reported Body Mass Index (BMI) and categorized as overweight and obesity according to World Health Organization (WHO) standards—overweight (25–29.9 kg/m²) and obesity (≥30 kg/m²)(1); and articles published in between 2015 and 2025.  Studies that assessed the prevalence and associated factors of overweight and obesity among type 2 diabetes mellitus patients primarily were took priority for inclusion. However, studies that did not primarily focus on overweight and obesity but reported relevant data and met the other inclusion criteria were also included. For analysis of associated factors, only studies reported statistical significant adjusted associations with corresponding 95% confidence intervals (CIs).  The exclusion criteria were: Reviews, editorials, commentaries, case reports, and conference abstracts without full data, studies published in languages other than English, animal researches, studies conducted in all types of diabetes which overweight and obesity were not reported separately for each type of diabetes. |  |
| Information sources | 6 | This was a systematic review and meta-analysis of studies reporting overweight and obesity among patients with type 2 diabetes mellitus in Ethiopia. A systematic and comprehensive searching of literature was conducted to identify studies reporting the prevalence of overweight and obesity and their determinants among patients with type 2 diabetes mellitus in Ethiopia. The databases searched were PubMed/MEDLINE, Scopes, Web of Sciences, and the Cochrane Library. In addition, grey literature was explored through Google and Google Scholar. Medical Subject Headings (MeSH) terms and relevant free-text keywords related overweight and obesity, type 2 diabetes mellitus, and Ethiopia.  . |  |
| Search strategy | 7 | The search strategy was guided by the CoCoPop framework consisting of (Co)-condition, (Co)-context and (Pop)- population(14): as (‘obesity’, ‘overweight’), (‘Ethiopia’), and (‘type 2 diabetes mellitus’) respectively. The article locating strategy was through (“overweight” OR “obesity” OR “body mass index” OR “BMI”) AND (“type 2 diabetes” OR “type II diabetes mellitus” OR “T2DM”) AND (“Ethiopia”). For studies that did not primarily focus on overweight and obesity we used “Metabolic Syndrome” OR “Metabolic Cardiovascular Syndrome” OR “Cardio-metabolic Syndrome” OR “Hypertension” OR ‘’High blood pressure’’ OR “Dyslipidemia” OR “Retinopathy’’ OR “Nephropathy” OR “Neuropathy” instead of (“overweight” OR “obesity” OR “body mass index” OR “BMI”). |  |
| Selection process | 8 | After the comprehensive literature search, all identified records were imported into the EndNote X9 reference management system software, and then duplicates were removed. Two independent reviewers (MJ, MK and TB) screened the remaining articles by using the settled inclusion criteria. Discrepancies between reviewers were resolved through discussion, and when necessary, other reviewers (SSA and HB) were consulted. |  |
| Data collection process | 9 | A second team of reviewers (BA and BT) evaluated the full-text articles for eligibility and methodological consistency before final inclusion. AN was the discrepancy resolver in this team during the study selection process. The predefined eligibility criteria, followed by documenting and reporting, were used for exclusion at the full-text screening stage. The overall process of study identification, screening, eligibility assessment, and inclusion was conducted in accordance with the PRISMA 2020 guidelines for systematic reviews and meta-analyses. |  |
| Data items | 10a | This search strategy aimed to identify all relevant published and unpublished primary studies related to the topic. |  |
|  | 10b | The selection was limited to studies published after the year 2015, in order to ensure that the most up-to-date and relevant studies were included. To identify additional eligible articles references list of included studies were also screened. |  |
| Study risk of bias assessment | 11 | In accordance with PRISMA 2020 recommendations the methodological quality of the included studies was critically appraised by using appropriate tool (JBI critical appraisal tool) to use when assessing questions of incidence by three independent authors (MJ, SSA and AAA)(15). The tool consists of 9 items designed to oversee the study population, sample size adequacy of the study, study subject and setting, reliability of condition or problem measurement, the appropriateness of the Statistical test used to analyze the data, and adequacy of the response rate of each selected article for the review process (Additional File Table S1).  Risk of bias was rated by questions of items that includes an answer of “Yes”, “No”, or “Unclear” to rate its risk of bias (ROB). The inclusion and exclusion of screened articles was based on the overall quality appraisal score in which an article was included scored equal or above to average (4.5) of the two independent authors (GTM and FAT). Any disagreement during scoring the articles between the involved authors was solved through the discussion and the third author (TB) was dispute them. |  |
| Effect measures | 12 | Two independent authors (DSN and SSA ) extracted the data by using a standardized data extraction manual developed in 2014 by Joanna Briggs Institute Reviewer(16). Author name, year of publication, sample size, study region, study design, prevalence and associated factors with overweight and obesity among type 2 diabetes and adjusted odds ratios with 95% confidence intervals were information extracted from studies. Any inconsistency was cross-checked and discrepancy raised during the data extraction process were resolved through discussion, and if consensus could not be reached, a third author (MT) made final decision. |  |
| Synthesis methods | 13a | All necessary and relevant information from every included study was extracted through Microsoft Excel spreadsheet, and then exported to STATA Version 17 for further analysis. The primary outcomes of interest were the pooled prevalence of overweight, obesity and overweight and obesity, as well as factors associated with overweight and/or obesity among persons with type 2 diabetes mellitus in Ethiopia. For each included study, the prevalence estimates and their corresponding standard errors were calculated. |  |
|  | 13b | The statistical heterogeneity among studies was assessed using Cochran’s Q test and quantified using the I² statistic, with I² values of 25%, 50%, and 75% indicating low, moderate, and high heterogeneity, respectively(17). |  |
|  | 13c | Subgroup analyses were performed based on study period, sample size, and study region to explore potential sources of heterogeneity. |  |
|  | 13d | Sensitivity analyses were conducted by sequentially removing individual studies to assess the robustness of the pooled estimates. |  |
|  | 13e | For factors associated with overweight and obesity among patients with type 2 diabetes mellitus were given a narrative synthesis and summary table, as most variables could not be quantitatively pooled due to heterogeneity in category. |  |
|  | 13f |  |  |
| Reporting bias assessment | 14 | Funnel plots and Egger’s regression asymmetry test were used to evaluate publication bias, with a visual inspection for the former and p-value <0.05 for the latter considered indicator of potential publication bias. |  |
| Certainty assessment | 15 |  |  |
| RESULTS | | |  |
| Study selection | 16a | A comprehensive literature search of PubMed/MEDLINE, Scopus, Web of Science, and the Cochrane Library identified **34,298 records**. In addition, **1,104 records** were identified through grey literature searches using Google and Google Scholar. After removal of **23,430 duplicate records** and exclusion of **6,134 records** based on title and abstract screening, **5,838 full-text articles** were assessed for eligibility. Of these, **52 studies** met the inclusion criteria and were included in the systematic review. Among them, **47 studies** were included in the meta-analysis of prevalence, and **6 studies** contributed data to the meta-analysis of associated factors. The study selection process is summarized using a PRISMA 2020 flow diagram (Figure 1). |  |
|  | 16b | Studies were excluded at the full-text screening stage for the following reasons: (i) review articles, editorials, commentaries, or conference abstracts without full data; (ii) studies conducted on mixed diabetes populations without separate reporting for type 2 diabetes mellitus; (iii) studies not reporting body mass index or not categorizing overweight and obesity according to WHO criteria; (iv) studies conducted outside Ethiopia; and (v) publications in languages other than English. Reasons for exclusion were documented and applied consistently according to predefined eligibility criteria. |  |
| Study characteristics | 17 | The review included **52 observational studies** published between **2015 and 2025**, comprising **47 cross-sectional**, **4 case–control**, and **1 prospective observational study**, with a total of **17,517 adult patients with type 2 diabetes mellitus**. Studies were conducted across multiple regions of Ethiopia, with the highest number from **Amhara (n=16)**, followed by **Oromia (n=11)**, **Addis Ababa (n=10)**, **SNNPR (n=5)**, **Tigray (n=4)**, **Sidama (n=3)**, and single studies from **Dire Dawa** and **Afar**. Five studies primarily assessed overweight and/or obesity, while the remaining studies reported BMI as part of broader metabolic or clinical assessments. Detailed characteristics are presented in **Table 1**. |  |
| Risk of bias in studies | 18 | The methodological quality and risk of bias of included studies were assessed using the **Joanna Briggs Institute (JBI) critical appraisal tool for prevalence studies**. Three independent reviewers conducted the appraisal. Studies scoring **≥4.5 out of 9** were included. Overall, the majority of studies demonstrated **low to moderate risk of bias**, with acceptable sampling methods, reliable BMI measurement, and appropriate statistical analyses. |  |
| Results of individual studies | 19 | For each included study, prevalence estimates of overweight, obesity, and combined overweight/obesity were extracted along with sample size and study setting.  Overall, 15 factors were reported to be significantly associated with overweight and obesity among patients with T2DM. These factors were grouped into three categories: socio-demographic factors (age, sex, place of residence, educational level, monthly income and family history of overweight/obesity); behavioral factors (physical activity/exercise, dietary practice, alcohol consumption, walking status, walking level and vigorous physical activity**); and clinical factors (comorbid hypertension, duration of diabetes, central obesity and triglyceride level)**. A summary of all factors associated with overweight and obesity is presented in Table 4. |  |
| Results of syntheses | 20a |  |  |
|  | 20b | Using a random-effects model:   - The pooled prevalence of **overweight** was 33.8% (95 CI: 30.7-37.0) (I² = 95.19%, p < 0.001) - The pooled prevalence of **obesity** was **17.1%** (95 CI: 13.6-20.6) (I^2^ =98.83%, p < 0.01) - The pooled prevalence of **combined overweight and obesity** was **51.1%** (95 CI: 45.9-56.3) (I^2^ =98.31%, p < 0.01) |  |
|  | 20c | Subgroup analyses were conducted by **study region**, **sample size**, and **study period**. Meta-regression analysis identified **study region** as the primary source of heterogeneity (p = 0.001), while study period and sample size were not statistically significant contributors. Regional variation was substantial, with the highest prevalence observed in **Addis Ababa**. |  |
|  | 20d | Sensitivity analyses were performed by sequentially excluding individual studies. The pooled prevalence estimates remained stable, indicating that no single study disproportionately influenced the overall results. |  |
| Reporting biases | 21 | Publication bias was assessed using the funnel plot and the regression-based Egger test for the test of small study effect. Although the funnel plot suggested asymmetry in visual inspection, the regression-based Egger test conducted under a random-effects model did not indicate any evidence of small study effects (β₁ = −2.46, SE = 4.51, z = −0.55, p = 0.585) |  |
| Certainty of evidence | 22 |  |  |
| DISCUSSION | | |  |
| Discussion | 23a | This systematic review and meta-analysis demonstrates that more than half of patients with type 2 diabetes mellitus in Ethiopia are affected by overweight or obesity, with substantial regional variation and higher prevalence in urban settings. Identified risk factors align with existing biological and behavioral evidence and highlight clustering of cardiometabolic risks. |  |
|  | 23b | Most included studies were cross-sectional, limiting causal inference. BMI was the sole measure of adiposity, which may misclassify body fat distribution. Several studies reported BMI as a secondary outcome, potentially affecting data quality. |  |
|  | 23c | Despite comprehensive searches, some relevant studies may have been missed. Residual heterogeneity remained after subgroup and meta-regression analyses. Only a limited number of studies were available for pooled analysis of associated factors. |  |
|  | 23d | The findings underscore the need for **integrated diabetes and weight management strategies**, particularly in urban areas. Policymakers and clinicians should prioritize lifestyle interventions, physical activity promotion, and targeted nutritional counseling. Future research should use longitudinal designs and alternative adiposity measures. |  |
| OTHER INFORMATION | | |  |
| Registration and protocol | 24a | The review protocol was registered in **PROSPERO** (Registration ID: **CRD1279684**). |  |
|  | 24b | The protocol is accessible through the PROSPERO database. |  |
|  | 24c | No major amendments were made to the registered protocol. |  |
| Support | 25 | No specific financial or non-financial support was received for this study. |  |
| Competing interests | 26 | The authors declare **no competing interests**. |  |
| Availability of data, code and other materials | 27 | All data extracted from included studies are presented within the manuscript and supplementary files. Analytic code and datasets are available from the corresponding author upon reasonable request. |  |

*From:*  Page MJ, McKenzie JE, Bossuyt PM, Boutron I, Hoffmann TC, Mulrow CD, et al. The PRISMA 2020 statement: an updated guideline for reporting systematic reviews. BMJ 2021;372:n71. doi: 10.1136/bmj.n71. This work is licensed under CC BY 4.0. To view a copy of this license, visit <https://creativecommons.org/licenses/by/4.0/>
