# Supplementary material for: Prevalence of overweight and obesity and associated factors among persons with type 2 diabetes mellitus in Ethiopia: A systematic review and meta-analysis
Source: Obes Pillars. 2026 Aug 8;19:100313. doi: 10.1016/j.obpill.2026.100313 (PMC13507751; doi:10.1016/j.obpill.2026.100313)
Supplement: Supplementary file 3 [file mmc3.docx]

**Table S 2: Methodological Quality assessment of the included primary studies**

Q1: Was the sample frame appropriate to address the target population?

Q2: Were study participants sampled appropriately?

Q3: Was the sample size adequate?

Q4: Were the study subjects and the setting described in detail?

Q5: Was the data analysis conducted with sufficient coverage of the identified sample?

Q6: Were valid methods used for the identification of the condition?

Q7: Was the condition measured in a standard, reliable way for all participants?

Q8: Was there an appropriate statistical analysis?

Q9: Was the response rate adequate and if not, was the low response rate managed appropriately?

| Authors name(Pub.date) | Q1 | Q2 | Q3 | Q4 | Q5 | Q6 | Q7 | Q8 | Q9 | Score/9 | |
| --- | --- | --- | --- | --- | --- | --- | --- | --- | --- | --- | --- |
| Daba Abdissa (2021) (22) | Y | Y | Y | Y | Y | Y | Y | Y | Y | 9 | |
| Kbrom Gemechu Kiros (2019)(18) | Y | Y | Y | Y | Y | Y | Y | Y | Y | 9 | |
| Temesgen Bizuayehu (2023)(19) | Y | Y | Y | Y | Y | Y | Y | Y | Y | 9 | |
| Eshetu Elfios Endrias (2021)(20) | Y | Y | Y | Y | Y | Y | Y | Y | Y | 9 | |
| Huzeyfa Kassaw(2024)(23) | Y | Y | Y | Y | Y | Y | Y | Y | UN | 8 | |
| Abera Lambebo Y(2018)(21) | Y | Y | Y | Y | Y | Y | Y | Y | Y | 9 | |
| Asamere Tsegaw (2021)(24) | Y | Y | Y | Y | Y | Y | Y | Y | Y | 9 | |
| Elias Mulat (2020)(25) | Y | Y | Y | Y | Y | Y | Y | Y | Y | 9 | |
| Aregash Abebayehu Zerga (2020)(26) | Y | Y | Y | Y | Y | Y | Y | Y | Y | 9 | |
| Worku Misganaw Kebede (2021)(27) | Y | N | Y | Y | Y | Y | Y | Y | Y | 8 | |
| Ginenus Fekadu (2019)(28) | Y | Y | Y | Y | Y | Y | Y | Y | Y | 9 | |
| Tewodros Yosef (2021)(29) | Y | Y | Y | Y | Y | Y | Y | Y | Y | 9 | |
| Gudisa Bereda (2021)(30) | Y | N | Y | Y | Y | Y | Y | Y | Y | 8 | |
| Rodas Getachew Abera (2022)(31) | Y | Y | Y | Y | Y | Y | Y | Y | Y | 9 | |
| Mohammed Gebre Dedefo (2020)(32) | Y | Y | Y | Y | Y | Y | UN | Y | Y | 8 | |
| Tadele Eticha (2016)(33) | Y | N | Y | Y | Y | Y | Y | Y | Y | 8 | |
| Berhane Fseha (2017)(34) | Y | Y | Y | Y | Y | Y | Y | Y | Y | 9 | |
| Zemenu Addis (2024)(35) | Y | N | Y | Y | Y | Y | Y | Y | Y | 8 | |
| Tsion Shibru (2019)(36) | Y | Y | Y | Y | Y | Y | Y | Y | Y | 9 | |
| Aster Wakjira Garedo (2024)(37) | Y | Y | Y | Y | Y | Y | Y | Y | Y | 9 | |
| Endalkachew Tedila Tarekegn (2025)(38) | Y | Y | Y | Y | Y | Y | Y | Y | Y | 9 | |
| Alemayehu Molla Tekalign (2025)(39) | Y | Y | Y | Y | Y | Y | Y | Y | Y | 9 | |
| Gebre Teklemariam Demoz (2019)(40) | Y | Y | Y | Y | Y | Y | Y | Y | Y | 9 | |
| Ali Seid Kolbay (2024)(42) | Y | Y | Y | Y | Y | Y | Y | Y | Y | 9 | |
| Gebrehiwot Lema Legese (2023)(43) | Y | Y | Y | Y | Y | Y | Y | Y | Y | 9 | |
| Yitagesu Mamo (2019)(44) | Y | Y | Y | Y | Y | Y | Y | Y | Y | 9 | |
| Yadelew Yimer Shibabaw (2023)(45) | Y | N | Y | Y | Y | Y | Y | Y | Y | | 8 |
| Biniyam Demisse Andarge (2025)(46) | Y | Y | Y | Y | Y | Y | Y | Y | Y | | 9 |
| Mohammed Hassen Salih (2024)(47) | Y | Y | Y | Y | Y | Y | Y | Y | Y | | 9 |
| Gebreamlak Gebremedhn Gebremeskel (2019)(48) | Y | Y | Y | Y | Y | Y | Y | Y | Y | | 9 |
| Agete Tadewos (2017)(56) | Y | Y | Y | Y | Y | Y | Y | Y | Y | | 9 |
| Abel Shita (2023)(58) | Y | Y | Y | Y | Y | Y | Y | Y | Y | | 9 |
| Belete Biadgo (2018)(49) | Y | Y | Y | Y | Y | Y | Y | Y | Y | | 9 |
| Girma Deshimo Lema (2024)(50) | Y | Y | Y | Y | Y | Y | Y | Y | Y | | 9 |
| Rukiya Debalke (2020)(51) | Y | Y | Y | Y | Y | Y | Y | Y | Y | | 9 |
| Mulatua Yeshitla (2025)(52) | Y | Y | Y | Y | Y | Y | Y | Y | Y | | 9 |
| Wabi Temesgen Atinafu (2025)(53) | Y | Y | Y | Y | Y | Y | Y | Y | Y | | 9 |
| Misganaw Asmamaw Mengstie (2024)(54) | Y | Y | Y | Y | Y | Y | Y | Y | Y | | 9 |
| Mitku Mammo Taderegew (2021)(55) | Y | Y | Y | Y | Y | Y | Y | Y | Y | | 9 |
| Firegenet Asnake Kitaw (2023)(57) | Y | Y | Y | Y | Y | Y | Y | Y | Y | | 9 |
| Kidist Tamru (2020)(59) | Y | Y | Y | Y | Y | Y | Y | Y | Y | | 9 |
| Kehabtimer S Kotiso (2020)(60) | Y | Y | Y | Y | Y | Y | Y | Y | Y | | 9 |
| Fikreab Desta (2024)(61) | Y | Y | Y | Y | Y | Y | Y | Y | Y | | 9 |
| Leteslase Hagos Gebreziher (2024)(62) | Y | Y | Y | Y | Y | Y | Y | Y | Y | | 9 |
| Haftay Gebremedhin (2020)(63) | Y | Y | Y | Y | Y | Y | Y | Y | Y | | 9 |
| Zemenu Addis (2025)(64) | Y | Y | Y | Y | Y | Y | Y | Y | Y | | 9 |
| Samuel Agegnew Wondm (2024)(65) | Y | Y | Y | Y | Y | Y | Y | Y | Y | | 9 |
| Alebachew Ferede Zegeye (2023)(66) | Y | N | Y | Y | Y | Y | Y | Y | Y | | 8 |
| Fasil Bayafers Tamene (2025)(67) | Y | Y | Y | Y | Y | Y | Y | Y | Y | | 9 |
| Tilaye Arega Moges (2025)(68) | Y | Y | Y | Y | Y | Y | Y | Y | Y | | 9 |
| Berhanu Kelemework (2024)(69) | Y | Y | Y | Y | Y | Y | Y | Y | Y | | 9 |
| Tsion Habtamu Ababiya (2023)(70) | Y | Y | Y | Y | Y | Y | Y | Y | Y | | 9 |

N=No, NA= Not Applicable, UN= Unclear, Y=Yes,
